# Engineering Gold Nanostructures for Cancer Treatment: Spherical Nanoparticles, Nanorods, and Atomically Precise Nanoclusters

**DOI:** 10.3390/nano12101738

**Published:** 2022-05-19

**Authors:** Wei He, Guanyu Ma, Quanli Shen, Zhenghua Tang

**Affiliations:** 1 Chongqing Chemical Industry Vocational College, Chongqing 401220, China; 2 Guangzhou Key Laboratory for Surface Chemistry of Energy Materials, New Energy Research Institute, School of Environment and Energy, South China University of Technology, Guangzhou Higher Education Mega Center, Guangzhou 510006, China; maguanyu1216@163.com (G.M.); sheng13005103058@163.com (Q.S.); 3 Guangdong Engineering and Technology Research Center for Surface Chemistry of Energy Materials, School of Environment and Energy, South China University of Technology, Guangzhou Higher Education Mega Center, Guangzhou 510006, China

**Keywords:** gold nanostructures, spherical nanoparticles and nanorods, atomically precise gold nanoclusters, cancer treatment, challenges and perspective

## Abstract

Cancer is a major global health issue and is a leading cause of mortality. It has been documented that various conventional treatments can be enhanced by incorporation with nanomaterials. Thanks to their rich optical properties, excellent biocompatibility, and tunable chemical reactivities, gold nanostructures have been gaining more and more research attention for cancer treatment in recent decades. In this review, we first summarize the recent progress in employing three typical gold nanostructures, namely spherical Au nanoparticles, Au nanorods, and atomically precise Au nanoclusters, for cancer diagnostics and therapeutics. Following that, the challenges and the future perspectives of this field are discussed. Finally, a brief conclusion is summarized at the end.

## 1. Introduction

Cancer is a worldwide health concern and one of the leading causes of mortality. In the past two decades, tremendous efforts have been dedicated to finding a competent treatment strategy against cancer, but only a few successes are achieved to date. Therefore, there is a huge demand for developing novel strategies for diagnostics and treatments of cancer. With the emergence and booming of nanoscience and nanotechnology, exceptional growth in research and applications of nanomaterials toward cancer treatment has been witnessed, bringing hope that the disadvantages of using conventional cancer therapies can be circumvented.

Among all kinds of nanomaterials for cancer treatment, gold nanostructures have shown great promise as emerging agents, mainly thanks to their unique advantages, such as tunable optical properties, easily functionalized surface, and excellent biocompatibility [1,2,3]. For instance, small gold nanoparticles are able to passively accumulate and remain at the tumor site through permeability and retention effects [4]. In addition, the surface of gold nanoparticles can be readily functionalized with active moieties such as peptides, proteins, monoclonal antibodies, and small drug molecules to avoid non-specific uptake and realize tumor-specific targeting [4]. Previous studies have shown that the structure of the gold nanomaterials can play a critical role. In an early comparative study of Au nanorods, nanocages, and nanohexapods for photothermal treatment, Au nanohexapods showed superior performance in both photothermal destruction and contrast-enhanced diagnosis [5]. In another investigation, Ma et al. evaluated the radio-sensitization effect in X-ray radiotherapy of three types of Au nanostructures (gold nanoparticles, spherical shape, AuNPs; gold nanorods, AuNRs; and gold nanospikes, AuNSs) and found that the efficiency of cellular internalization followed the order AuNPs > AuNSs > AuNRs [6].

It is worth noting that gold nanostructures include various types, and from the size and morphology, plus the consideration of employing in biomedical research, in this review, three major types of gold nanostructures, namely spherical gold nanoparticles, gold nanorods, and atomically precise gold nanoclusters, for cancer treatment are discussed. Notably, the three gold nanostructures have some common yet differentiated advantages for curing cancers, and some of the common merits have been mentioned above. Generally, upon illumination, spherical gold nanoparticles and gold nanorods have localized surface plasmon resonance (LSPR) which is attributed to the oscillations of free electrons [7]. The LSPR is very sensitive to the size, morphology, capping agent, and refractive index on the surface, making the optical absorption of the gold nanospheres and nanorods range from visible to near-infrared. However, for gold nanoclusters, such LSPR phenomenon disappears; instead, discrete absorbance peaks can be observed [8,9]. Additionally, gold nanoclusters can be synthesized with molecular purity with determined composition and defined structure, which offers a perfect platform for building structure–functionality relationships in biomedical research [10,11].

**Scheme 1:**
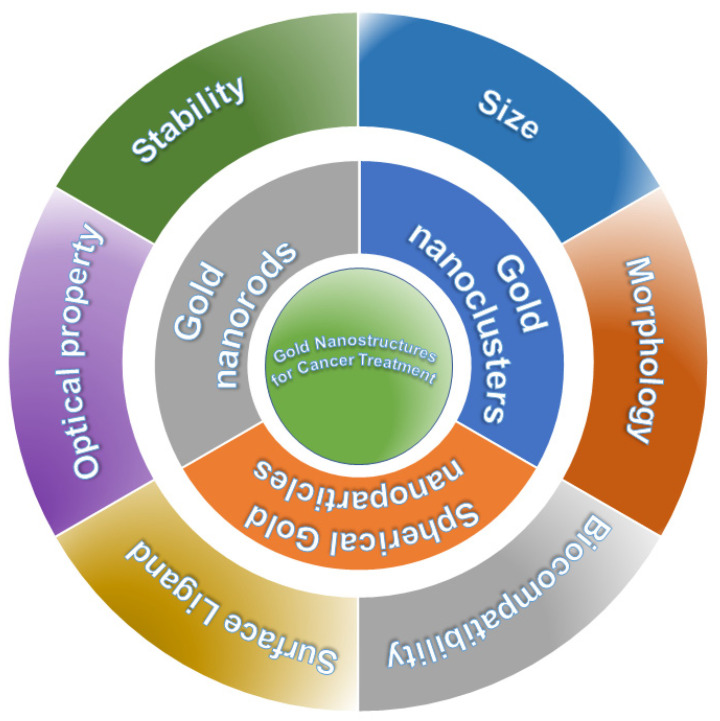
Gold nanostructures for cancer treatment.

In this review, the recent progress regarding employing spherical gold nanoparticles, gold nanorods, and atomically precise gold nanoclusters for cancer treatment is discussed first (Scheme 1). The main factors, including size, morphology, optical property, surface ligand, biocompatibility, and stability, that can affect the performance will be particularly mentioned. Following that is the elaboration of the challenges and perspectives in the field, and this review will be ended with a summarized outlook and conclusion. As gold nanostructures for cancer treatment are extensively covered in the literature and many related reviews can be found [12,13,14,15,16,17,18], this review is limited to the following scope: (1) In terms of gold nanostructure shape, it only focuses on spherical gold nanoparticles, gold nanorods, and gold nanoclusters. (2) In terms of cancer type, it only targets high-incidence cancers such as breast cancer, liver cancer, skin cancer, and colon cancer. (3) Finally, for cancer treatment types, it mainly focuses on photothermal/photodynamic therapy, drug carrier/delivery system, cellular imaging, and biosensing/probing, despite there being some differences for different shaped gold nanostructures.

## 2. Gold Nanostructures for Cancer Treatment

### 2.1. Spherical Gold Nanoparticles

Spherical Au nanoparticles have been widely utilized in cancer treatment, mainly thanks to their unique physicochemical properties, their excellent biocompatibility, and particularly their characteristic surface plasmon resonance absorbance with specific wavelengths [19,20]. Spherical Au nanoparticles can be applied for cancer treatment in several ways such as photothermal therapy, radiotherapy, tumor imaging, and serving as drug delivery systems [21,22,23,24,25,26].

Note that for photothermal cancer treatment, AuNP-based agents can convert the light-irradiation energy into heat and generate reactive oxygen species for ablating the tumor cells [27,28,29]. The AuNPs can be delivered by physiological transportation or conjugation with antibodies, and once they are delivered, they can self-assemble into large clusters inside the cells [30]. For instance, Emami et al. reported the construction of anti-PD-L1-targeting and doxorubicin (DOX)-conjugated Au nanoparticles (PD-L1-AuNP-DOX) for the targeted chemo-photothermal therapy of colorectal cancer [31]. The results showed that PD-L1-AuNP-DOX treatment plus NIR irradiation drastically and synergistically inhibited the proliferation of CT-26 cells in vitro by increasing apoptosis and cell cycle arrest [31]. In another study, Mao and Yang’s group took advantage of the electrostatic attraction and successfully assembled negatively charged silk fibroin (SF) with positively charged gold nanoparticles (AuNPs) [32]. The in vitro and in vivo analysis revealed that the AuNPs/SF nanofibers can kill breast cancer cells and destroy the tumor issues under just one-time NIR irradiation for 6 min by photothermal therapy efficiently [32]. In addition, Wang et al. discovered that hollow microporous carbon and Au nanoparticles can be integrated as well to synergistically increase the photothermal conversion effect while ensuring a high DOX loading capacity [33]. In addition, AuNPs can act as a gatekeeper to release DOX at a specific location and promote the photothermal effect [33].

**Figure 1:**
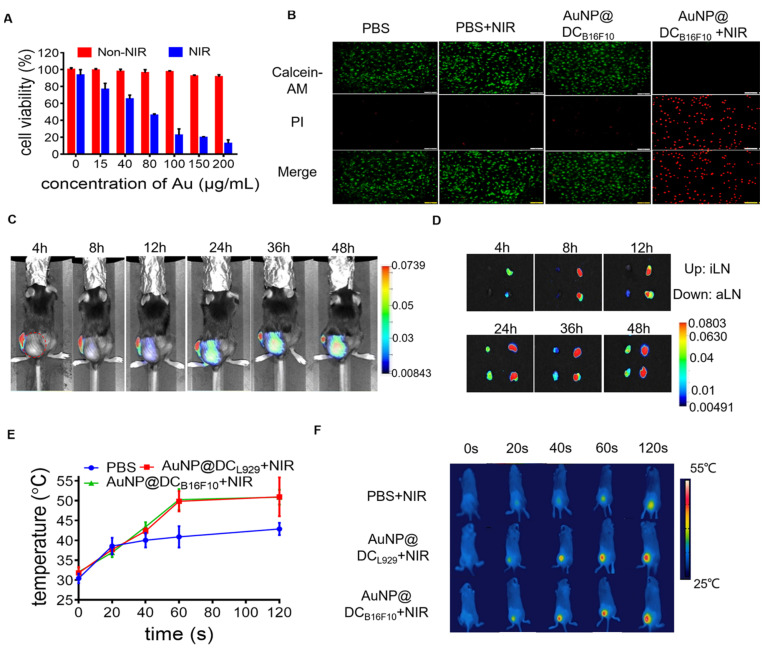
Cytotoxicity and characterization of AuNP@DCB16F10. (**A**) Cell viability of B16F10 cells treated with AuNP@DCB16F10 after incubation for 24 h. (**B**) Cytotoxicity images of B16F10 cells. (**C**) In vivo distribution of AuNP@DCB16F10 labeled by DiR in tumor-containing mice. (**D**) Luminescence images of nanoparticle migration. (**E**,**F**) Infrared thermal images and temperature profiles of tumor-containing mice with injection of PBS, AuNP@DCL929, and AuNP@DCB16F10. Reprinted with permission from [34]. Copyright 2019, American Chemical Society.

By taking advantage of the great biocompatibility, enhanced permeability and retention effect, and the plasmonic optical properties, one typical strategy for using gold nanoparticles to treat cancer is photo-induced hyperthermia and immunotherapy [34]. As a typical example, in 2019, Zhang and co-workers reported the use of a type of novel immunological AuNPs via intra-cellular generation and exocytosis for combinatorial photothermal therapy and immunotherapy. Melanoma B16F10 cells were utilized to produce AuNPs first and subsequently shed nanoparticle encapsulated vesicles that were transported to an extracellular location by tumor antigens (AuNP@B16F10) [35]. When NPs were introduced into dendritic cells (DCs), DC-derived AuNPs (AuNP@DCB16F10) were generated. As illustrated in Figure 1A, laser irradiation showed that the phototoxicity of nanoparticles was concentration-dependent and the cell viability was extremely low, suggesting that AuNP@DCB16F10 can effectively kill tumor cells. In addition, the cells treated with nanoparticles or laser alone did not die, while AuNP@DCB16F10 + NIR showed full red fluorescence with barely green fluorescence (Figure 1B). Figure 1C presents the fluorescence images at different time spacings upon the injection of AuNP@DCB16F10, and the fluorescence intensity of the tumor increased and reached the maximum at 24 h. AuNP@DCB16F10 was able to drain to inguinal lymph nodes quickly, then rapidly to axillary lymph nodes on the same flank with the injection spot, and then slowly drained to the nanoparticles to the opposite lymph nodes when the fluorescence intensity increased over time (Figure 1D). As shown in Figure 1E,F, upon irradiation, the temperature of AuNP@DCB16F10-treated mice increased quickly from 32 °C to over 42 °C in the first 40 s and reached 50 °C after 60 s. This confirmed that AuNP@DCB16F10 was able to convert light to hyperthermia in vivo very efficiently [35].

Furthermore, spherical gold nanoparticles can also self-assemble into three-dimensional nanostructures for cancer treatment. In a recent study, You’s group reported nanoprobes formed by self-assembly of ultrasmall AuNPs for dual-mode real-time imaging-guided photothermal/photodynamic combined therapy for liver cancer [36]. The nanoprobes demonstrated excellent tumor-targeting capabilities in both T1 magnetic-resonance imaging and in vivo fluorescence imaging modes; meanwhile, the nanoprobes are able to dissociate and escape from the body to reduce aggregation in the body to minimize the possible toxicity [36].

In addition, spherical Au nanoparticles have been attracting considerable interest as non-toxic drug carrier systems for cancer treatment, thanks to the large surface-to-volume ratio; easy tuning of surface charge, hydrophilicity, and functionality; and outstanding stability [37,38,39]. Various biocompatible polymers (e.g., polyethylene glycol (PEG) [40], polyelectrolyte [37], DNA [25], liposome [41], and other bio-macromolecules [42]) can be used to tune the tumor microenvironment [43] and, more importantly, enhance the stability, payload capacity, and the cellular uptake. Muhammad et al. reported that the PEG-capped AuNPs can enable efficient delivery of anti-cancer therapeutics of bleomycin and doxorubicin into HeLa cells while maintaining drug cytotoxicity [40]. In another study, Soliman’s group successfully prepared cetyltrimethylammonium bromide (CTAB)-stabilized AuNPs which can efficiently entrap fluorouracil (5-FU), an antimetabolite drug used for treating colon and skin cancers [44]. The optimum 5-Fu-loaded AuNP gel and cream were able to reduce tumor volume by about 6.8- and 18.4-fold, as compared to the control, in A431-bearing mice [44].

### 2.2. Gold Nanorods

Another important type of gold nanostructure is gold nanorods, which possess some unique advantages for cancer treatment. For example, gold nanorods can absorb light in the near-infrared (NIR) region, enabling efficient irradiation, which can be utilized for selective photothermal therapy of some specific cancers [45]. Specifically, thanks to the tunable localized surface plasmon resonance (LSPR), gold nanorods can not only serve as probes but also become heat sources when irradiated by a laser with a photothermal effect [46]. The generated heat can provide photothermal therapy for cancer treatment and/or trigger anti-cancer drug release for chemotherapy when gold nanorods serve as a drug carrier [46]. In short, gold nanorods can be applied for cancer treatment in phototherapy, cellular imaging, drug transport, and combined therapy (e.g., phototherapy and chemotherapy) [47,48].

Employing the photothermal effects of gold nanorods to kill cancer cells is the most widely employed strategy for cancer treatment, as the nanorod can absorb the NIR light to penetrate into sick tissues without damaging the surrounding healthy tissues, and the wavelength of light can be fine-tuned through the aspect ratio and surface ligand [49,50,51]. In 2015, Betzer et al. reported dual-mode targeted plasmonic nanoprobes made of gold nanorods as a theranostic approach for detecting and curing skin-adjacent tumors for head and neck cancers [52]. Both in vivo and in vitro, the immune-targeted gold nanorods can target head and neck cancer cells with high specificity and facilitate the differentiation between cancerous and noncancerous tissues [52]. Shrivastava’s group discovered that the polyelectrolyte coating on the Au nanorods can have an important effect on the photothermal efficiency and the photothermally triggered cancer cell damage [53]. For gold nanorods with polystyrene sulfonate (PSS-AuNRs) and PSS plus poly-diallyl dimethyl ammonium chloride (PDDAC-AuNRs), despite high photothermal conversion efficiency and cellular uptake of PDDAC-AuNRs, their intracellular clustering adversely affects the photothermal treatment of cancer cells [53]. Such surface coating influence was also observed by Wang et al., who documented biologically inspired polydopamine-stabilized Au nanorods for light-induced cancer therapy [54]. The self-polymerized polydopamine shell has a high adsorption capacity for therapeutic drugs and is very stable and biocompatible. Thanks to the tunable LSPR properties of gold nanorods in the near-infrared spectral region, impressive in vitro cancer cell killing efficiency and remarkable tumor growth suppression were achieved in vivo by the gold nanorod–polydopamine composite, superior to any single therapy modality [54].

Besides surface coating, imprinting other biologically active molecules such as saccharides can also improve the photothermal treatment efficiency. Liu’s group prepared sialic acid (SA, a typical monosaccharide)-imprinted gold nanorods, which could selectively kill a tumor but not damage the circumjacent healthy tissue [55]. Besides achieving higher treatment efficiencies, researchers have also devoted great effort to unraveling the molecular mechanism of the Au-nanorod-aided plasmonic photothermal therapy. In 2017, Ali et al. conducted an investigation regarding the efficacy, toxicity, and mechanism of Au nanorod photothermal therapy of cancer in xenograft mice [56]. In this study, the size, surface modification, and concentration of AuNRs and the laser power to achieve the maximal apoptosis induction were first examined. The possible mechanism of AuNRs-plasmonic photothermal therapy (PPTT) action using quantitative proteomic analysis in tumor tissues of the mouse was also studied, where several death pathways were identified. Cytochrome c and p53-associated apoptosis mechanisms were recognized to contribute to the enhancement of PPTT with AuNRs@RF (rifampicin). Moreover, Pin1 and IL18-related signaling made a contribution to the disturbance of the NETosis pathway through PPTT enabled by AuNRs@RF [56].

**Figure 2:**
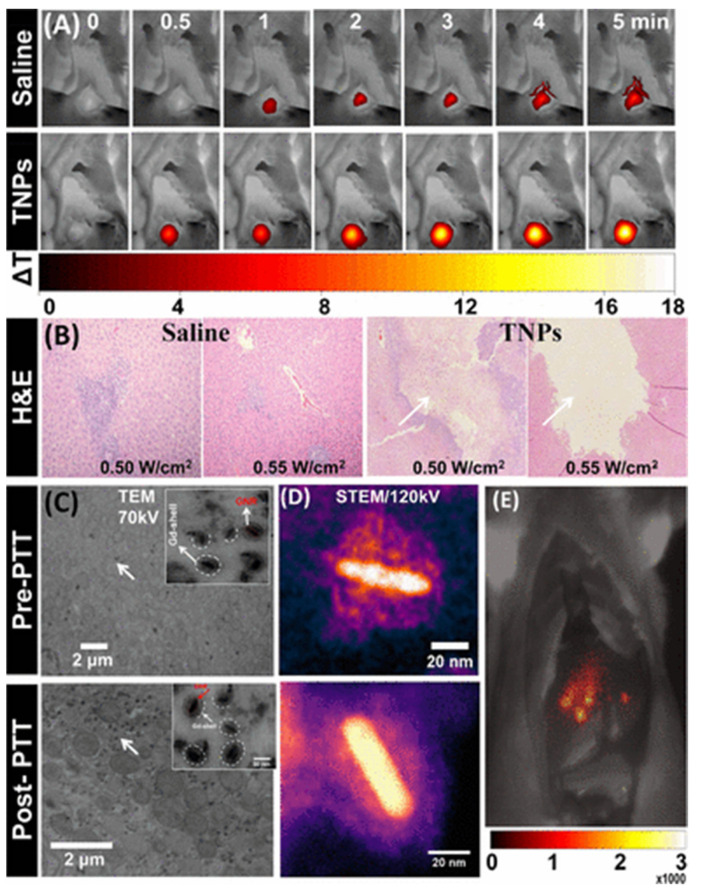
In vivo photothermal therapy of rats. (**A**) Thermal images of CRLM tumor-containing rats. (**B**) Typical histological images of saline and TNP-ablated tumor tissue stained with H&E. Pre-PTT and post-PTT tissue images recorded using (**C**) TEM at 70 kV and (**D**) STEM at 120 kV. (**E**) Emission monitoring after injecting 50 μL of TNP solution (excitation: ~980 nm, emission: ~1550 nm). Reprinted with permission from [57]. Copyright 2018, American Chemical Society.

In 2018, Joshi’s group reported gold-nanorod-composed theranostic nanoparticles (TNPs) for interventional image-directed photothermal therapy for solid tumors [57]. In this study, the feasibility of site-selective hepatic image-directed delivery of TNPs in rats was examined. Figure 2A shows the dynamic thermal imaging at different time points during the PPT process. In the saline group, the tumor’s temperature increased by about 7.5 °C within 1 min and remained basically stable; however, in sharp contrast, the TNP group tumor temperature quickly jumped to ~20 °C in 5 min, suggesting that the increase in tumor temperature exceeded the range of hyperthermia, resulting in the damage of local vasculature which can destroy the tumor cells effectively. The authors further conducted the hematoxylin/eosin staining of tumor sections. As shown in Figure 2B, tumor slices in the saline group exhibited no obvious effect, while the TNP group presented a valid response under the same laser irradiation power level with a remarkable photothermal therapy effect. The transmission electron microscopy (TEM) images verified that the TNPs stayed in the tissue with no structural change, as illustrated in Figure 2C. In addition, the clear observation of the morphology of a gold nanorod core and a Gd shell can be observed in Figure 2D. Finally, Figure 2E validates the feasibility of intraoperative imaging-offered quantum yield, and the imaging sensitivity can be further improved by reducing the exposure time to below 1 s. The above findings confirm that TNPs can be employed for photothermal ablation efficiently while bearing no risk of heat-induced breakdown [57].

Meanwhile, gold nanorods can integrate with other functional materials such as inorganic compounds to form a therapeutic package to further promote the efficiency of cancer treatment. Note that a variety of organic photosensitizer-conjugated Au complexes have been designed and prepared recently, but they also have some drawbacks such as photobleaching and invalid energy transfer, and the introduction of inorganic compounds might resolve these issues. For instance, Lee et al. fabricated novel inorganic phototherapeutic complexes by conjugating Au nanorods with defective TiO_2_ nanoparticle clusters together [58]. A higher efficacy of cell death was observed in phototherapeutic treatments of cancer cells, which is attributed to the increase in reactive oxygen species generation from the TiO_2_ nanoparticle clusters with the aid of localized surface plasma resonance triggered electron and heat generation from Au nanorods [58]. In another study, Li et al. fabricated a novel nanocomposite of mesoporous silica gold nanorods, which also showed an improved lifetime of circulation and homotypic targeting to HeLa cell tumors [59]. By utilizing this nanocomposite, the tumor growth can be completely inhibited, indicating great potential for tumor treatment [59].

Besides the photothermal effects, gold nanorods (GNRs) can serve as effective drug carriers for controllable drug delivery. For instance, Mahmoud and co-workers discovered that cholesterol-coated gold nanorods can be an intriguing carrier for hydrophobic drugs, where efficient delivery and therapy against breast cancer cells can be achieved by using MCF-7 cell lines [60]. A quite recent study quantified the cellular uptake by GNRs in MCF-7 cells by using inductively coupled plasma mass spectrometry, and the MCF-7 cells used the micropinocytosis mechanism to internalize bare GNRs that aggregate and associate with the cell membrane [61]. Pacardo et al. discovered that when functionalized with cyclodextrin, gold nanorods can encapsulate doxorubicin (DOX), and the as-formed nanocomplex showed enhanced anti-cancer efficacy [62]. Zhang et al. reported DNA-conjugated gold nanorods as a multifunctional carrier, which can load and release DOX at targeted locations [63]. More importantly, such biotin-PEG-functionalized GNR nanomedicine was able to drastically increase the cell uptake and reduce the drug reflux capability of multidrug-resistant breast cancer cell lines [63].

One may notice that more and more research attention has been switched to employing gold nanorods and/or gold-nanorod-based nanomedicines for combined therapies, especially chemotherapy and photothermal therapy, as combined chemo-photothermal therapy shows better therapeutic efficiency than monotherapy. For instance, in 2014, Wang et al. reported combined chemotherapy and photothermal ablation using DOX-loaded DNA-wrapped gold nanorods for the treatment of metastatic breast cancer [64]. The inhibition capability of tumor growth was mainly thanks to the synergistic effect between DOX-induced apoptosis and laser-irradiation-caused necrosis of tumor cells [64]. In 2019, the Qian and Suo groups developed a facile means to construct polysaccharide-encapsulated Au nanorods for improved chemo-phototherapy of breast cancer [65]. The polysaccharide-decorated nanoplatform was efficiently internalized inside MCF-7 breast cancer cell lines and exhibited greater cancer cell killing than single modalities [65]. Recently, Huang et al. prepared pH-sensitive gold nanorods conjugated with a polypeptide for chemo/photothermal therapy for cervical cancer treatment [66]. The Au nanorod conjugates displayed exceptional biocompatibility, improved cancer cell uptake, and excellent cancer cell killing effects [66]. Another recent study conducted by Zhu’s group further demonstrated that degradable silica-capped gold nanorods can be employed for triple-combined therapy for breast cancer treatment [67]. Specifically, in the nanomedicine, upon 808 nm laser irradiation, singlet oxygen was generated to achieve photodynamic/photothermal effects, while the site-specific drug release of DOX can realize chemotherapeutic outcomes [67].

### 2.3. Atomically Precise Gold Nanoclusters

Gold nanoclusters (AuNCs), usually with a size less than 3 nm, are intermediate bridges between relatively larger plasmonic Au nanoparticles and Au complexes. A gold nanocluster has tens to a few hundreds of gold atoms, possessing a core–shell structure, with Au atoms in the core and a surface ligand capped on the metal core. For biomedical applications, various biomolecules, such as DNA, proteins, polypeptides, dendrimers, and biopolymers, have been employed as the stabilizing ligand to prevent the aggregation of the metal core and hence improve the stability. Thanks to the ultrasmall-size-imparted quantum confinement effects, gold nanoclusters exhibit significantly different optical behaviors and chemical and catalytic properties compared with their nanoparticle counterparts [9,68,69]. Unlike AuNPs, AuNCs have no surface plasmon resonance absorption peak but have discrete absorption peaks ranging from the visible region to the near-infrared (NIR) region and drastically different fluorescent properties, depending on the size, surface ligand, charge state, and other factors. Tremendous efforts and progress have been made in employing AuNCs for cancer treatments, and the main ways AuNCs can make a contribution include probing, cell imaging, photothermal therapy, radiotherapy, and antimicrobial application [70,71,72,73].

By rational structural design and choosing of a surface ligand, AuNCs can be fluorescent at a specific photo-emitting wavelength with a long lifetime that is quite favorable for imaging or as probes [73]. In 2017, Singh developed glucose-decorated Au nanoclusters as membrane-potential-independent fluorescence probes that can realize rapid identification of cancer cells that express the Glut receptor [74]. In another study, Chen et al. fabricated novel iodinated gold nanoclusters stabilized by bovine serum albumin (BSA) as a dual modality probe, which achieved malignant thyroid cancer visualization through fluorescence/computed tomography (CT) [75]. Wang’s group discovered that accurate tumor imaging can be realized by gold nanoclusters conjugated with carborane derivatives, making accurate imaging-guided cancer treatment possible [76]. Such cancer imaging behaviors were also observed by Zhu et al., who prepared gold-nanocluster-grafted polymer nanoparticles for both imaging and cancer cell killing [77]. Phototherapy is usually considered to be a more powerful means to cure cancer. For example, Liu et al. found that dendrimer-encapsulated Au nanoclusters can “self-supply” O_2_ through the catalase activity, which was utilized for photodynamic therapy to overcome cancer hypoxia [78]. In another report, Youn’s group designed a facile top-down approach to synthesize albumin/polyallylamine-assisted AuNCs, which possessed a non-spherical and hyperbranched morphology with a high absorption capacity [79]. Such structure advantage was favorable for surface-plasmon-based hyperthermia, and hence the as-fabricated gold nanoclusters were markedly cytotoxic to 4T1 breast cancer cells [79]. Recently, more and more research attention has been devoted to employing AuNCs in radiotherapy, in which ionizing radiation is utilized for killing cancer cells. Zhang et al. prepared histidine-capped gold nanoclusters that can be adopted as a radiosensitizer for improved cancer radiotherapy through synergistic internal and external regulations [80]. Interestingly, Yang’s group found that radionuclide-labeled gold nanoclusters, particularly 99mTc@AuNCs and 177Lu@AuNCs, were able to boost the effective anti-tumor immunity for augmented cancer radiotherapy [81]. Li’s group employed bone marrow mesenchymal stem cells to mediate the fabrication of ultrasmall gold nanoclusters, which can enhance the radiotherapy efficacy of Egr1-hNIS for its radiation sensitization [82]. In another report, Li and co-workers demonstrated a transformable AuNC aggregate-based synergistic strategy, which can improve the tumor retention/penetration of the nano-radiosensitizers and weaken the radio-resistance of cancer cells [83]. In a quite recent study, Burda’s group and Basilion’s group reported that when conjugating AuNCs with protease activatable monomethyl auristatin E, the specificity and efficacy of radiation and chemotherapy can be significantly improved [84]. Both in vitro and in vivo results showed selective tumor cell uptake, excellent anti-tumor activity, and prolonged chemotherapeutic effect [84].

It is worth noting that gold nanoclusters with polydisperse size distribution are employed in the above cases. Such wide size distribution can hinder the deeper fundamental understanding of biomedical applications to some extent. However, gold nanoclusters of molecular purity can be chemically synthesized with atomic precision. Atomically precise gold nanoclusters have demonstrated great potential for cancer treatment, mainly due to their rich surface functionalities, outstanding optical features (especially the excellent luminescent properties, e.g., strong emission in the near-infrared region), and great biocompatibility [10,85,86,87]. More importantly, thanks to the definite size, uniform composition, and crystallographically resolvable structure, atomically precise gold nanoclusters provide an ideal platform to unravel comprehensive mechanisms and establish structure–activity relationships in cancer treatment study [88,89,90].

In early studies, biocompatible compounds such as glutathione (GSH) were widely employed as functional stabilizing agents to prepare atomically precise gold nanocluster molecules [91]. For instance, Zhang et al. synthesized a series of ultrasmall molecular Au_10-12_(SG)_10-12_ nanoclusters, which enhanced the tumor uptake and targeting specificity via enhanced permeability and retention effects owing to their small-size-imparted quantum confinement effect. At the same time, GSH ligands can further enhance the tumor uptake by facilitating the escape of nanoclusters from the reticuloendothelial system while activating the transporter [92]. Such size-depending tumor-targeting behaviors were subsequently observed by Zheng and co-workers with a series of few-atom AuNCs [93]. Upon injection into the mice for 40 min, smaller-sized Au_10-11_ and Au_18_ NCs were more retained in the kidneys than the relatively larger-sized Au_25_ NCs. Additionally, the ratios of bladder-to-kidney intensity followed the order of Au_25_ NC > Au_18_ NC > Au_10-11_ NC. This suggests that the glomerulus is no longer a one-way “size-cutoff” slit but is an atom-precise “bandpass” barrier that can drastically decrease the renal clearance of atom-precise Au nanoclusters in the subnanometer size regime [93]. In a following study, the same group reported that enhanced photostability and tumor-targeting can only be achieved by ICG-conjugated GSH-protected Au_25_ nanoclusters but not gold clusters with other gold numbers [94]. Such magic size selection was observed by Liu group in a recent study, in which the Au_25_(Capt)_18_-based nanosystem acted as a GSH-activated mitochondria-targeting photosensitizer for high-efficiency treatment of malignant tumors [95].

**Figure 3:**
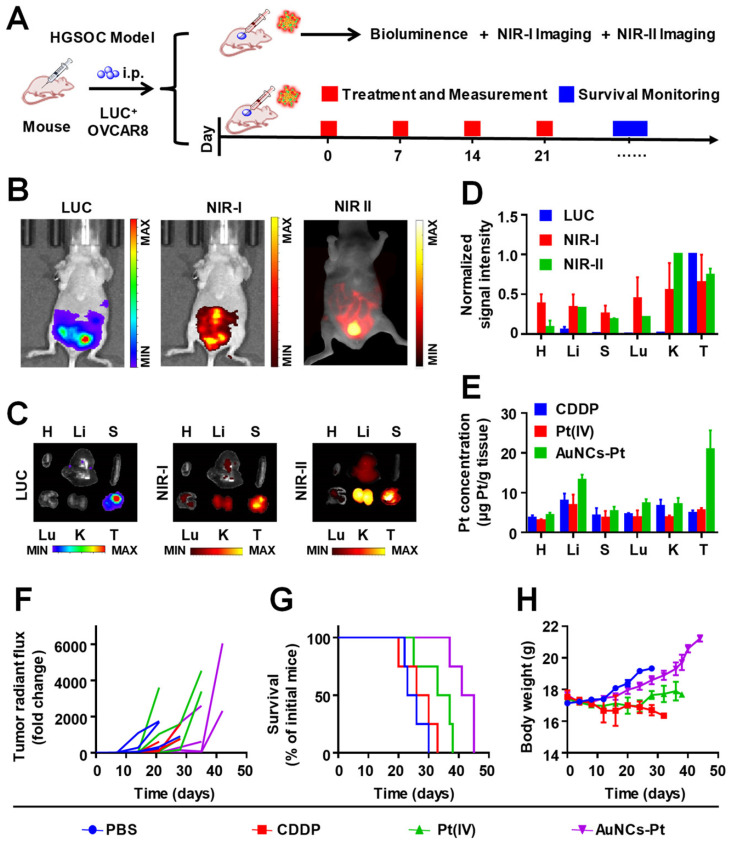
In vivo imaging and therapeutic efficiency of Au_25_NCs-Pt on an orthotopic model. (**A**) Scheme of establishing an orthotopic model. (**B**) Representative in vivo images at 12 h after AuNCs-Pt administration. (**C**) Ex vivo images and (**D**) the intensities of ex vivo signal in heart (H), liver (Li), spleen (S), lung (Lu), kidney (K), and tumor (T) at 12 h. (**E**) Pt distribution after 12 h injection. (**F**) Time duration of the tumor burden. (**G**) Survival of the HGSOC-bearing mice. (**H**) Body weights of mice during the treatment. Reprinted with permission from [96]. Copyright 2020 American Chemical Society.

In 2020, Yang et al. developed a theranostic nanomedicine of AuNCs-Pt based on atomically precise glutathione-protected Au_25_ nanoclusters with dual functions of both near-infrared imaging and glutathione scavenging capabilities [96]. AuNCs-Pt has NIR-II (excited at 808 nm, emitted at 1050–1250 nm) imaging ability on a lethal high-grade serous ovarian cancer (HGSOC) model; hence, it can be a potential tool for monitoring Pt transportation [96]. At the same time, AuNCs-Pt exhausts the intracellular glutathione to minimize the Pt detoxification and effectively maximizes the platinum chemotherapeutic efficacy [96]. As shown in Figure 3A, the authors conducted NIR imaging using the LUC + OVCAR8 cells. Notably, LUC + OVCAR8 cells have a bioluminescent property that is able to present the growth degree and position of tumors through imaging. After injection for 12 h, most of the AuNCs-Pt was found in the peritoneal tumor, indicating high tumor accumulation (Figure 3B). It is worth noting that the images in the NIR-I region portrayed the tissue anatomy. In stark contrast, the NIR-II signal was better defined and overlapped with the tumor luminescent signal. Ex vivo imaging was carried out on excised organs, which verified the colocalization of the bioluminescent and fluorescent signals of both NIR-II and NIR-I for the AuNCs-Pt tumor deposits (Figure 3C,D). Thanks to the stronger penetration capability, NIR-II imaging more precisely disclosed the nanoparticle accumulation in organs, showing a more convincing imaging method. The results indicated that AuNCs-Pt reached about 5-fold Pt accumulation in tumor tissue compared with that using free CDDP (Figure 3E). They also illustrated that AuNCs-Pt demonstrated a markedly stronger ability to inhibit tumor growth compared to the other groups (Figure 3F). Furthermore, AuNCs-Pt treatment increased the survival of the animals to one and a half months and did not reduce the body weight (Figure 3G,H).

**Scheme 2:**
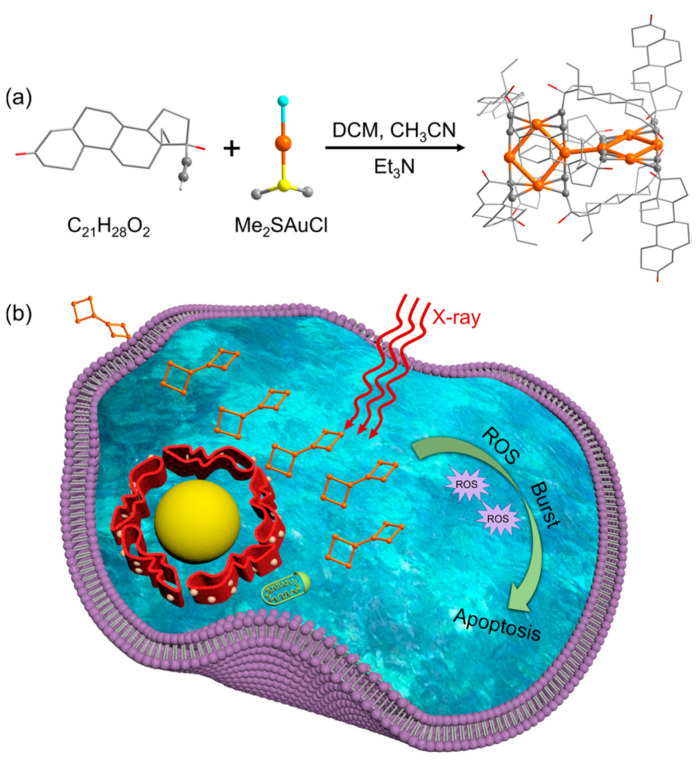
(**a**) The synthetic scheme of Au_8_NCs; color code: orange—Au, red—O, yellow—S, turquoise—Cl, gray—C. Hydrogen atoms are omitted for clarity. (**b**) Au_8_NCs for cancer radiotherapy. Reprinted with permission from [100]. Copyright 2019, American Chemical Society.

The above case took full advantage of the near-infrared emission property of molecular Au_25_ nanoclusters, and the Au_25_ clusters can effectively maximize the chemotherapeutic efficacy of platinum. In fact, besides chemotherapy, radiotherapy is another important cancer therapeutic strategy, particularly for treating solid tumors at different stages [97]. In radiotherapy, X-ray radiation of high energy is used to shrink the tumors and kill cancer cells, and the radiosensitizer is essential to improve the therapeutic efficacy [98,99]. In 2019, Jia et al. reported a molecular levonorgestrel-protected gold nanocluster as a radiosensitizer for enhanced cancer therapy [100]. Scheme 2a presents the synthetic route, in which the alkynyl ligand of levonorgestrel can react with Me_2_AuSCl to generate a molecular Au_8_ nanocluster. Single crystal X-ray diffraction (SCXRD) measurement showed that it has two parts, each containing a planar tetranuclear structure capped by four ligands. The major cancer therapeutic mechanism is shown in Scheme 2b. Specifically, X-ray irradiation triggers an increase in reactive oxygen species, leading to irreversible cell apoptosis. Au_8_NCs make cancer cells more sensitive to radiation by improving the local treatment efficiency with a relatively safe and low radiation dose.

**Figure 4:**
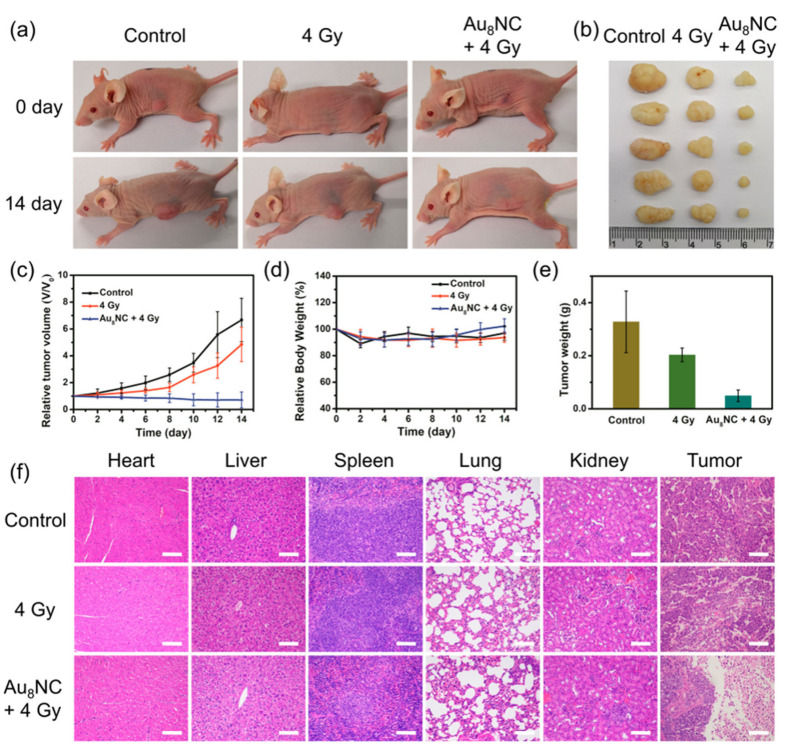
In vivo tumor assay of Au_8_ nanoclusters. (**a**) Typical images of mice under different conditions at 0 and 14 days. (**b**) Photos of dissected tumors. (**c**) Relative tumor volume curves. (**d**) The curves of relative mice body growth. (**e**) The tumor weights. (**f**) H&E histological staining of excised organs and tumor slices. X-ray irradiation energy: 6 MV. Reprinted with permission from [100]. Copyright 2019, American Chemical Society.

The authors then evaluated the radiosensitizing effect of the Au_8_ nanoclusters with an in vivo tumor assay [100] in which a comparison test of a control sample and a phosphate-buffered saline (PBS)-treated group was also performed. Specifically, the EC1 cells were first divided into three different groups: control group, PBS-treated group, and Au_8_NC-treated group. Subsequently, EC1 cells (2 × 10^6^ cells per mouse) were injected into the flanks of female BALB/c-nude specific-pathogen-free (SPF) mice and treated with different doses of X-ray irradiation. Finally, the body weights and the tumor sizes were monitored every other day [100]. Figure 4a–e illustrate the tumor size and body weight of the mice after injection of different doses. It can be noted that an approximately 5 times increase in the tumor size was observed for the control groups, while in sharp contrast, the tumor volume in the Au_8_NCs + 4 Gy group decreased significantly. Furthermore, the body weights of the mice under various conditions remained nearly the same over 2 weeks, indicating no toxicity. Eosin and hematoxylin staining of the organs and tumors was further carried out. As shown in Figure 4f, compared with the control groups, ubiquitous damage can be identified in the tumor tissue for the Au_8_NCs + 4 Gy treated group with basically no abnormalities in the organs. This study demonstrated the potent capability of the atomically precise gold nanoclusters as a sensitizer to enhance the tumor-suppressing efficacy. Following the above work, the same group also reported a levonorgestrel-protected gold nanocluster of Au_10_(C_21_H_27_O_2_)_10_; by conjugating a poly(allylamine hydrochloride) molecule, sustained drug release and effective antibody-mediated actin imaging can be realized [101]. We also notice that the ligand of levonorgestrel is a water-soluble drug, and this study can pave a path to selecting a suitable drug as a ligand to prepare molecular Au nanoclusters as effective sensitizers for improved radiotherapy and beyond.

## 3. Challenges and Perspectives

The recent advances regarding gold nanostructures, namely gold nanoparticles, gold nanorods, and atomically precise gold nanoclusters, have been reviewed above. One can notice that the gold nanostructures hold great potential in cancer diagnostics and therapeutics, mainly thanks to the merits such as excellent optical properties, facile control of size and/or morphology, robust stability, the capability to tune the surface chemistry for conjugation with functional biological molecules, and especially the great biocompatibility.

However, there are also some disadvantages of gold nanostructures employed for cancer treatment, and these disadvantages need further in-depth investigations in this promising yet fast-evolving field:

1. The long-term toxicity issue. The gold nanostructures cannot be easily degraded and can accumulate in vivo during prolonged treatment, which may cause some uncertain side effects [56,102]. Upon long-term accumulation, damage to organs such as lung, spleen, kidney, and liver might be present.
2. The targeting specificity issue. Even though the gold nanostructures can be designed to bind to specific cancer cells, there is still an urgent need for cancer diagnosis and therapeutics at the early stage with a high level of targeting specificity [103]. Currently, the widely employed cancer treatment strategies such as photoimaging and photothermal therapy still have the limitations such as non-specific binding and the unnecessary activation of the normal host immune response.
3. The modulation of the gold nanostructures to meet the complex biological environment can be challenging. Upon the surface modification of the gold structures, the pharmacokinetic parameters of the gold nanostructures and the cellular response will be correspondingly changed, while in vivo, the fundamental comprehensive understanding of the interactions between the gold nanostructures and the biological moieties is still lacking [104].
4. Some gold nanostructures (e.g., the gold nanocluster case mentioned in this review) can be used for both NIR-I and NIR-II imaging; however, when choosing both regions, the excitation wavelength range is quite limited, and the imaging effectiveness and efficiency still have room to improve. Determining how to modify the composition, morphology, and structure of these gold nanomaterials to work better for both NIR-I and NIR-II regions is still extremely challenging.

The above challenges actually imply great opportunities for future development using gold nanostructures for cancer treatment. In addition, from the perspectives of this research field, some other important issues may also represent the future research directions:

1. For photothermal treatment based on gold nanostructures, the efficacy is highly dependent on the penetration depth of the NIR lasers, and the heating intensity can decrease with the increase in the laser penetration depth. This means that the laser intensity and the plasmonic effects of the gold nanostructures could be critical and deserve special attention in future studies.
2. Even if gold nanostructures have been successfully documented for in vitro, in vivo, pre-clinical, and clinical studies, considering the cytotoxicity, the internalization of gold nanostructure with tissues, the complex biological environment, the long-term stability of the gold nanostructure’s integrity, and the high costs of preparing specifically designed nanogold agents, the way to realizing gold nanostructures for practical applications of cancer treatment is still long.

However, all the above issues or challenges might be resolved by the rapid development of nanotechnology, plus other factors such as the introduction of artificial intelligence in modern medicine. For instance, with the aid of artificial intelligence and machine learning technologies, some new specific drugs can be possibly designed and synthesized for preparing atomically precise gold nanoclusters to target specific cancer cells to achieve some “perfect” diagnostic and therapeutic effects.

## 4. Conclusions

In conclusion, gold nanostructures, especially spherical gold nanoparticles, gold nanorods, and atomically precise gold nanoclusters, are good candidates for cancer treatment. The optical properties (such as surface plasmon effects and fluorescent behaviors), ease of surface modification, low cytotoxicity, outstanding biocompatibility, excellent stability, and other merits make gold nanostructures very promising for cancer diagnostics and therapeutics. Despite some shortcomings and disadvantages, we envision that more research endeavors will push gold nanostructures toward real clinical applications of cancer treatment in the future.
